# Effect of vestibular rehabilitation games in patients with persistent postural perceptual dizziness and its relation to anxiety and depression: prospective study

**DOI:** 10.1007/s00405-023-08369-z

**Published:** 2023-12-21

**Authors:** Nadia Mohamed Kamal Ibrahim, Nagwa Mohamed Abdelmonem Hazza, Dhiaa Mohammed Yaseen, Eman Mohamed Galal

**Affiliations:** 1 Audiology Unit, Otorhinolaryngology Department, Faculty of Medicine, Ain Shams University, Cairo, Egypt; 2 Hearing and Balance Center, Basra Teaching Hospital, Basra, Iraq

**Keywords:** Vestibular rehabilitations therapy, Persistent postural perceptual dizziness, Posturography, Anxiety, Depression

## Abstract

**Purpose:**

To evaluate the efficacy of vestibular rehabilitation therapy (VRT) for management of patients with persistent postural perceptual dizziness (PPPD) utilizing subjective and objectives outcome measures and to study the effect of degree of both anxiety and depression in patients on the response of vestibular rehabilitation therapy.

**Methods:**

Thirty-three PPPD patients participated in this study. Selection of patients was based on the diagnostic criteria for PPPD stated by Barany society in the International Classification of Vestibular Disorders (2017). Every patient was subjected to history taking, anxiety and depression assessment, Arabic version of Dizziness Handicap Inventory (DHI), and sensory organization test (SOT). All patients received vestibular rehabilitations therapy. Assessment of VRT outcome was conducted after 6 weeks of VRT.

**Results:**

The mean patients’ age was 40.9 ± 16.3 years, and nearly equal gender distribution. Vestibular migraine was the most precipitating condition (24.2%) in patients with PPPD. (39.4%) of patients had abnormal scores of anxiety and depression tests, all patients had from moderate to severe degrees of handicap caused by dizziness as measured by DHI, most of patients had abnormal findings in all conditions of SOT. After vestibular rehabilitation therapy, DHI and SOT scores showed significant improvement after VRT. More improvement was found among the group with no anxiety and depression.

**Conclusion:**

VRT were effective in improving balance abnormalities in patients with PPPD evidenced by subjectively by DHI scores and objectively by SOT results. PPPD patients with concomitant psychiatric disorders; anxiety and depression experienced the least degree of improvement.

## Introduction

Bárány society (2017) has grouped previously named conditions; visual vertigo (VV), space and motion discomfort (SMD), phobic postural vertigo (PPV), and chronic subjective dizziness (CSD) together under the umbrella term “Persistent Postural-Perceptual Dizziness PPPD” and has given their diagnostic criteria. PPPD may be precipitated by conditions that disrupt posture or cause vertigo, unsteadiness, or dizziness, including peripheral or central-vestibular disorders, other medical illnesses, or psychological distress [1]. No specific laboratory test for PPPD is available, and the precise assessment of symptoms, exacerbating factors, and medical history are essential for PPPD diagnosis [2].

PPPD is classified as a chronic functional vestibular disorder; it is not a structural or psychiatric condition. PPPD cannot be attributed to a specific structural lesion within the vestibular system, but is rather a maladaptive dysfunction of postural control and central-vestibular processing. PPPD alone does not produce evidence of active vestibular dysfunction [1].

The exact pathophysiological mechanisms remain unclear; failure of re-adaption related cortical overexcitability of the vestibular system after neuro-otologic diseases, functional changes in postural control mechanisms, cortical spatial integration, or other dizziness-related conditions all seem to play a crucial role for the development and chronification of PPPD [3].

The high rate of psychiatric disorders comorbidity in patients with PPPD was reported. Generally, Furman et al. [4] and Goddard et al. [5] found that the onset and interaction between vestibular disorders and psychiatric disorders is attributed to the overlapping central nervous system transmission of the vestibular and mood information pathways. The vestibular nucleus has many nerve fiber projections with mood-related nuclei, such as the parabrachial nuclei, the locus coeruleus, and the dorsal raphe nuclei, and also interacts with the frontal lobe, hippocampus, and dentate gyrus. This affects the release of neurotransmitters, causing dysfunction in these mood-related regions and affecting the development of anxiety and depression.

Vestibular rehabilitation therapy (VRT), cognitive–behavioral therapy (CBT), and antidepressant medications were proposed as treatment modalities for patients with PPPD. However, the mechanisms of antidepressant medication have not yet elucidated, and the level of evidence is low [6].

VRT can help patients escape a cycle of maladaptive balance control, recalibrate vestibular systems, and regain independence in everyday life [6]*.* There is a growing literature supporting the effectiveness of vestibular rehabilitation as a treatment option for patients with PPPD [7]*.* Customized VRT adequately reduce symptoms and improve quality of life in subjects with PPPD [8]*.* VRT is being found essential not only to promote optimal recovery but also to prevent more refractory engrained PPPD [9]*.* Accordingly, this research aimed at studying the effectiveness of VRT in PPPD patients and its relation to the degrees of anxiety and depression in these patients.

## Materials and methods

### Materials

This study was conducted in Basrah Hearing and Balance Center in Iraq. Using Power Analysis and Sample Size Software (PASS 15) (Version 15.0.10) for sample size calculation, done by medical research ethical committee of faculty of medicine; the setting power at 80%, alpha error 0.05, and after reviewing previous thesis results, a sample size of at least 30 patients diagnosed with persistent postural perceptual dizziness was needed. The research ethics committee approval was on 1\11\2020, No. FWA00017585.

Selection of study group was based on the diagnostic criteria for PPPD proposed by Barany society shown in the International Classification of Vestibular Disorders (ICVD) 2017 [1] (Appendix). Severely ill patients who would not tolerate VRT were excluded.

### Methods

Every patient was subjected to:

1. History taking:

Detailed history taking emphasizing on the symptoms elaborated in diagnostic criteria by Barany society for PPPD ICVD (2017). It involved the full account of the patients’ character of dizziness (dizziness which is non-motion sensations of disturbed or impaired spatial orientation, unsteadiness which is feelings of being unstable while standing or walking, non-spinning vertigo which is distorted sensations of swaying, rocking, bobbing, or bouncing of oneself or the surroundings), the frequency (need not to be present continuously throughout the day, present most days, for 3 months or more), duration (hours long), progression of symptoms (may wax and wane) and the exacerbated factors; upright posture, active or passive motion without regard to direction or position and exposure to moving visual stimuli or complex visual patterns. Also, the history of acute, episodic, and chronic vestibular disorder was taken in addition to review of systems, past medical history (neurologic or psychiatric, etc.), and past drugs history [1].

2. Anxiety and depression assessment by the Hospital Anxiety and Depression Scale (HADS)

The Hospital Anxiety and Depression Scale (HADS) is a 14-item measure designed to assess anxiety and depression symptoms in medical patients, with emphasis on reducing the impact of physical illness on the total score. The depression items tend to focus on the anhedonic symptoms of depression (absence of enjoyment, motivation, and interest). Items are rated on a 4-point severity scale. The HADS produces two scales, one for anxiety (HADS-A) and one for depression (HADS-D), differentiating the two states. Scores of greater than or equal to 11 on either scale indicate an abnormal case [10].

**Scoring:** Total score: Depression (*D*) ________ Anxiety (*A*) ________

0–7 = Normal

8–10 = Borderline abnormal (borderline case)

11–21 = Abnormal (case).

3. Arabic version of Dizziness Handicap Inventory (DHI)

DHI was translated in Arabic language by Al-Gohary et al [11]*.* All patients were asked to answer this questionnaire, which consisted of 25 questions. These questions were designed to evaluate the patient`s dizziness functionally, physically, and emotionally. The scoring involved the following: (yes) response was given 4 points; (sometimes) response was given 2 points and 0 point for (no). Total maximum possible score was 100% for a significant self-perceived handicap, 0% suggested no handicap. In attempt to evaluate the degree of handicap, a score up to 25% considered mild handicap, 25–50% was moderate handicap, 50–75% was moderately severe, and > 75% was severe handicap [12]. The evaluation was conducted at the onset (baseline) and immediately at the end of sessions of VRT.

4. **Sensory organization test (SOT) using** Computerized Dynamic Posturographic Synapsys system\Marseill, France, SN 3923000055.

All the patients were evaluated using the Sensory Organization Test that monitors the three sensory systems involved in maintaining balance (proprioceptive, visual, and vestibular). The evaluation was conducted at the onset (baseline) and immediately at the end of sessions of VRT.

5. Vestibular rehabilitations therapy (VRT): All patients received.

**5.1 Clinic-based VRT:** Supervised sessions three times per week for 6 weeks using smart dynamic posturography systems.

The VRT program consisted of standardized rehabilitation exercises for all patients, these exercises were included in the software of the posturography system; sessions were scheduled three times per week for 6 weeks. Each session began with a test reference which helped monitor the patient’s evolution throughout the rehabilitation. The difficulty level of vestibular rehabilitation sessions was individualized to each patient`s performance; throughout the rehabilitation sessions, the training level was gradually and adaptively raised based on the patient's performance. By reducing the period of time required to complete the games, adding foam to the posturography platform to reduce somatosensory cues, and changing the visual background to follow the patient's movements, the exercises became harder. The standardized rehabilitation sessions included games as four groups: Stabilization, Weight shift, Weight bearing, and Postural control.

**5.1.1 Stabilization exercises:**

- *Simple stabilization* The patient will be instructed to stay as stable as possible inside a holding area for a fixed duration (the games are calibrated based on the patient limit of stability).
- *Stabilization with stress* The patient will be asked to keep his balance despite the presence of a disruptor (stress), if the disruptor touches the patient, the time remaining inside is reset to zero.

**5.1.2 Weight shifting and weight-bearing exercises:**

In weight shifts, the patient must reach a target by performing a weight shifting then come back to the initial position, while in weight-bearing, the patient must perform a weight shifting then stay stable in this position.

**5.1.3 Postural control exercises:** the patient was trained to maintain balance in games that combine the stabilization, the weight shift and the weight-bearing exercises; 3D tunnel exercise, the goal of the game is to arrive at the end of the tunnel and 2D Maze exercise where the patient must reach the end of the maze.

**5.2 Home-based VRT:**

It aimed to improve gaze and postural stability, done for 15 min 2 times per day and for 6 weeks, namely (VOR X1) and (Walk with head movement exercise) [13].

6. Assessment of VRT outcome was conducted by DHI and SOT both before VRT and after 6 weeks of VRT sessions (immediately after the last session of VRT).

### Data management and analysis

Data were tabulated and statistically analyzed using SPSS, version 20 (SPSS Inc., Chicago, IL, USA). Quantitative data were described as mean and standard deviation (minimum–maximum). Qualitative data were expressed in frequencies and percentage. Specific tests (mentioned later) used for comparing data.

## Results

The mean patient age for current study was 40.9 ± 16.3 (12–68) years, and nearly equal gender distribution of men 17 (51.5%) and women 16(48.5%).

**Fig. 1:**
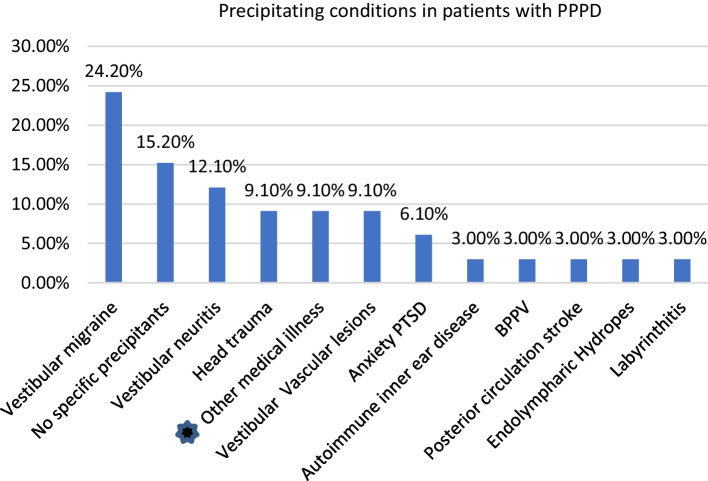
Distribution of precipitating conditions for patients with PPPD. Other medical conditions (one case of adrenal gland disorders Addison’s disease and 2 cases of fibromyalgia)

Figure 1 shows that vestibular migraine was the highest precipitating condition in patients with PPPD, while condition of nonspecific precipitants ranked as the 2nd. Vestibular disorders account as 54.4% of cases.

Table 1 shows that about (39.4%) of patients had abnormal scores of anxiety and depression tests.

**Table 1:** Distribution of patients according to Hospital Anxiety and Depression Scale

Degree	Anxiety assessment	Depression assessment
	no. (%)	no. (%)
Normal	12 (36.4%)	12 (36.4%)
Borderline	8 (24.2%)	8 (24.2%)
Abnormal	13 (39.4%)	13 (39.4%)
Mean ± SD (min–max)	9.4 ± 3.9 (3–17)	9.2 ± 3.7 (3–17)

Table 2 reveals that all patients had ranged from moderate to severe degrees of handicap caused by dizziness as measured by DHI.

**Table 2:** Distribution of patients according to the DHI scores

Degree of handicap (DHI scores)	Score range	*N* (%)
Mild	0–25%	0 (0)
Moderate	25–50%	4 (12.1%)
Moderately severe	50–75%	11 (33.3%)
Severe	> 75%	18 (54.5%)

### Wilcoxon signed-rank test

Tables 3 illustrates that Dizziness Handicap Inventory Index (DHI) score showed significant reduction after VRT especially in the physical domain.

**Table 3:** Comparison between Dizziness Handicap Inventory Index (DHI) domain scores before and after VRT

DHI domains	Before VRT	After VRT	*P*
	Mean ± SD	Mean ± SD	
Functional	69.2 ± 14.6	23.8 ± 16.2	0.000*
Emotional	69.4 ± 14.5	24.9 ± 12.9	0.000*
Physical	95.6 ± 124.5	24.2 ± 10.6	0.000*
Total score	70.5 ± 12	23.2 ± 9.4	0.000*

**Fig. 2:**
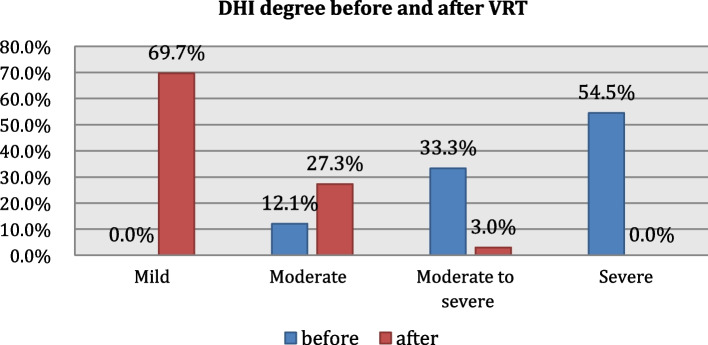
Distribution of DHI degrees before and after VRT

Table 3 and Fig. 2 illustrate that Dizziness Handicap Inventory Index (DHI) score showed significant reduction in the handicap after VRT.

Table 4 shows that most of patients had abnormal findings in all conditions of Sensory Organization Tests (SOT), regarding sensory analysis the vestibular system showed the highest percentage of abnormality.

**Table 4:** Distribution of PPPD patients in relation to sensory organization tests (SOT) scores of computerized dynamic posturography (CPD) both anteroposterior (AP) and mediolateral scores (ML)

SOT	Anteroposterior (AP)	Mediolateral (ML)
	*N* (%)	*N* (%)
Abnormal C1	15 (45.5%)	18 (54.5%)
Abnormal C2	18 (54.5%)	28 (84.8%)
Abnormal C3	29 (87.9%)	25 (75.8%)
Abnormal C4	16 (48.5%)	24 (72.7%)
Abnormal C5	30 (90.9%)	23 (69.7%)
Abnormal C6	29 (87.9%)	26 (78.8%)
Abnormal global score	29 (87.9%)	31 (93.9%)
Abnormal somatosensory	19 (57.6%)	15 (45.5%)
Abnormal visual	16 (48.5%)	12 (36.4%)
Abnormal vestibular	29 (87.9%)	25 (75.8%)
Abnormal visual preferential	17 (51.5%)	18 (54.5%)

Table 5 shows statistical significant improvement in SOT scores (both AP and ML) after VRT.

**Table 5:** Comparison between Sensory Organization Tests (SOT) scores before and after VRT

SOT		Before VRT	After VRT	*P*		Before VRT	After VRT	*P*
		Mean ± SD (min–max)	Mean ± SD (min–max)			Mean ± SD (min–max)	Mean ± SD (min–max)	
C1	Anteroposterior (AP)	84.8 ± 6.6 (62–93)	89.1 ± 2 (84–94)	0.001*	Mediolateral (ML)	89.5 ± 3.4 (80–96)	92.6 ± 1.3 (89–96)	0.000*
C2		78.2 ± 9.1 (54–91)	85 ± 3.5 (78–92)	0.000*		85.3 ± 5.1 (72–94)	91 ± 2.1 (84–95)	0.000*
C3		57.4 ± 15.5 (25–83)	72.9 ± 9 (43–87)	0.000*		67.9 ± 14.6 (20–86)	79.5 ± 7.1 (56–90)	0.000*
C4		66.2 ± 13.4 (31–89)	74.9 ± 7 (50–88)	0.000*		70.5 ± 9.9 (44–87)	79.6 ± 5.7 (69–90)	0.000*
C5		44.1 ± 12.6 (15–72)	61 ± 7.4 (41–74)	0.000*		52.8 ± 14 (19–77)	72.6 ± 6.9 (52–85)	0.000*
C6		25.2 ± 11.4 (8–63)	39.1 ± 12.6 (12–67)	0.000*		37.1 ± 12.8 (12–67)	54 ± 12.4 (32–78)	0.000*
Global score		50.2 ± 13 (29–74)	70.6 ± 6.6 (56–86)	0.000*		59.8 ± 12.1 (26–80)	78.6 ± 4.2 (70–87)	0.000*

Paired *t* test was used, *P* value < 0.05 is considered statistically significant

Tables 5 and 6 show that there is statistically significant difference between before and after VRT as regards all tests of anteroposterior (AP) and mediolateral (ML) aspects of Sensory Organization Tests (SOT) by computerized dynamic posturography (CPD) where most of patients showed functional improvement.

**Table 6:** Comparison between sensory analysis scores (AP and ML) before and after VRT

Sensory analysis (AP and ML)		Before VRT	After VRT	*P*
		Mean ± SD (min–max)	Mean ± SD (min–max)	
Somatosensory	AP	85.9 ± 10 (62–100)	94.4 ± 4.4 (81–100)	0.001*
Visual		78.6 ± 15.9 (31–100)	86.9 ± 6.9 (70–100)	0.010*
Vestibular		44.7 ± 17.3 (10–84)	71.2 ± 12.3 (40–96)	0.000*
Visual preferential		65.7 ± 19.9 (16–92)	76.6 ± 10.7 (51–96)	0.000*
Somatosensory	ML	92.5 ± 8.6 (65–100)	98.3 ± 1.9 (90–100)	0.000*
Visual		83.6 ± 10.5 (53–100)	88.1 ± 5 (76–100)	0.000*
Vestibular		60.4 ± 14.8 (20–81)	80.2 ± 8.8 (55–92)	0.000*
Visual preferential		65.4 ± 19.6 (22–93)	80.1 ± 9.6 (55–96)	0.000*

Paired *t* test was used; *P* value < 0.05 is considered statistically significant

Table 7 shows overall significant difference between anxiety and depression degrees as regards the improvement in global AP and ML score of SOT, with more improvement among the group with normal anxiety and depression scores.

**Table 7:** Comparison between mean differences of AP and ML global score of SOT before and after VRT among the different degrees of anxiety and depression

Mean difference in global score between before and after		*N*	Mean ± SD	Min–max	Overall *P*	*P* (a and b)	*P* (b and c)	*P* (a and c)
*Among anxiety levels*								
Global AP score	Normal^a^	12	27.92 ± 13.20	4.0–46.0	0.001*	1.000	0.008*	0.001*
	Borderline^b^	8	26.50 ± 5.71	15.0–34.0				
	Abnormal^c^	13	9.77 ± 12.08	− 3.0 to 35.0				
Global ML score	Normal^a^	12	25.25 ± 10.30	6.0–37.0	0.000*	1.000	0.003*	0.001*
	Borderline^b^	8	25.13 ± 9.39	14.0–44.0				
	Abnormal^c^	13	9.00 ± 9.81	1. 0–31. 0				
*Among depression levels*								
Global AP score	Normal^a^	13	26.31 ± 14.19	− 1.0 to 46.0	0.011*	1.000	0.070	0.017*
	Borderline^b^	7	25.71 ± 9.11	7.0–34.0				
	Abnormal^c^	13	11.69 ± 12.22	− 3.0 to 35.0				
Global ML score	Normal^a^	13	25.15 ± 10.23	6.0–37.0	0.003*	1.000	0.054	0.004*
	Borderline^b^	7	22.86 ± 11.44	10.0–44.0				
	Abnormal^c^	13	10.31 ± 10.75	1.0–31.0				

One-way ANOVA test and Bonferroni post hoc test was used, *P* value ≤ 0.05 is considered statistically significant

*SD* standard deviation, *N* number per group, *Min* minimum, *Max* maximum

## Discussion

The definition of PPPD underlines the importance of precipitating events that initiate the syndrome, where the diagnostic criteria stated that this disorder is precipitated by vestibular insults, neurological or medical illness. In the current study, the vestibular insults represented (57.4%) of cases; the most common cause was vestibular migraine (24.2%), followed by the vestibular neuritis (12%) (Fig. 1). On the other hand, the percentage of PPPD patients with no specific precipitants was (15.2%). Yagi et al. [14] found the high levels of PPPD symptoms in nonclinical populations were because PPPD is a spectrum that pre-exists in the population rather than just being a consequence of vestibular insult. Also, it was demonstrated that PPPD can develop primarily on its own, without somatic triggers, or secondary to an organic disorder [15].

Accordingly, PPPD is not a disorder of the vestibular periphery; rather, it is considered a functional disorder caused by shifts in the functioning of spatial orientation systems to favor visual or somatosensory/proprioceptive stimuli over vestibular inputs.

From this study, we found that (39.4%) of the patients also exhibited abnormally high levels of anxiety and depression measured by HADS as shown in Table 1. Also, Maslovara et al*.* [16] noticed that the 40% of PPPD group had pathological anxiety and 23% exhibited pathological levels of depression. This high percentage of anxiety and depression found among patients with PPPD is attributed to neural interactions between central-vestibular pathways and neural networks of anxiety and fear [17]*.*

The majority of PPPD patients (87.7%) revealed moderate-to-severe and severe degrees of handicap measured by the Arabic version of the Dizziness Handicap Inventory (DHI) (Table 2). Therefore, the presented symptoms compromise the self-perception of body balance and interfere with the quality of life of these patients. Additionally, the majority of the patient's functional, physical, and emotional scores ranged from moderate to severe disability. This is consistent with the outcomes of Maslovara et al. [18]. Again, Sui and Prepageran reported that most of PPPD patients exhibited a moderate level of disability [19].

In this study, the program of rehabilitation on the posturography included two main goals; postural stability and gaze stabilization in the form of virtual reality games. Among the sensory components of VRT, visual stimuli using virtual reality environments are not only enjoyable but also effective in PPPD, where the visual stimuli are closely related to the symptoms of PPPD. There was statistically significant improvement in the DHI questionnaire post-vestibular rehabilitation therapy in the three domains of DHI; physical, functional, and emotional domain (Table 3). According to Jacobson [19], a change in DHI scores of 18 or more was considered a significant improvement, and DHI showed significant improvement by 47.3 points in our study (mean total DHI scores before VRT was 70.5, while after VRT, the mean total DHI scores was 23.2). Also, most of the patients turned to mild and moderate degrees of handicap after VRT instead of moderate-to-severe degrees before VRT (Fig. 2). These results were in agreement with the results of Nada et al.’s study [8] that showed a significant decrease in functional, physical, and total scores on the DHI in patients with PPPD after VRT.

It was noted from Table 4 that SOT abnormality was found in all conditions (C1, 2, 3, 4, 5, and 6) with different degrees of abnormality. The higher percentage of abnormality were in conditions C5 (90.9%) and C6 (87.9%), indicating primarily functional vestibular dysfunction and the incorrect use of vestibular cues. Also, the abnormalities in the rest of conditions (1, 2, 3, and 4) give idea about large variability between the six SOT conditions without a specific pattern. Söhsten et al. [20] demonstrated that the belief of poor scores on lower numbered conditions lack a pathophysiologic basis and have to be re-considered for patients with PPPD. This could be caused by a confluence of three sensory inputs and posturography results consistent with brain mechanisms involved in PPPD and should not be misinterpreted as evidence of malingering. Therefore, patients with PPPD had difficulties with postural control across multiple sensory challenges, consistent with postulated neurophysiologic mechanisms of this condition.

Also, there was statistically significant improvement after VRT for all the sensory analysis scores of SOT (Table 6). This was in agreement with the other studies [21, 22].

Because of the uncertainty in the pathogenesis of PPPD, at present, no clear mechanism of action has been established for either pharmacological or non-pharmacological interventions. In a trial to find the current evidence for non-pharmacological treatments for PPPD, a review done by Webster et al. [23] concluded that there is currently no evidence from randomized controlled trials to support a 'gold standard' treatment for PPPD, although some interventions are in widespread use [23].

Regarding vestibular rehabilitation therapy, previous studies used variable methods and programs for vestibular rehabilitation; no specific program has been settled. In Seo et al.’s study [24], they used customized vestibular exercise and optokinetic stimulation using a virtual reality system with a head mounted display once a week for 4 weeks. They concluded that vestibular rehabilitation can improve dizziness, quality of life, and gait function in PPPD.

Table 7 shows that there is overall statistically significant relation between the degrees of anxiety or depression and the mean difference between before and after VRT global CDP ML & AP scores. It was also noted that subjects with normal anxiety and depression scores experienced better improvement of SOT scores after VRT. Although the group with abnormal scores of anxiety and depression did not achieve as high outcomes as those who do not report symptoms of psychological distress, this group may need more rehabilitation sessions or may need additional medical treatment and psychiatric consultation.

By far, the most common psychiatric comorbidity in vertigo patients is anxiety and/or depression. The psychiatric state of the patient plays an important role in the prognosis of vertigo disease [25], but is often neglected by clinicians, which affects the efficacy of vertigo treatment. Both psychological and physical factors must be considered in the clinical treatment of vertigo, and the psychological status of patients warrants increased attention.

## Conclusion

Using CDP used as objective methods to assess the postural control improvement after VRT and the DHI questionnaire as a subjective outcome measure post-vestibular rehabilitation therapy, give stronger evidence of reliability of unit of assessment in this study.

This study provided evidenced effectiveness of vestibular rehabilitation using smart CDP systems to reduce symptoms in patients with PPPD taking into consideration the degree of anxiety and depression as these patients may need more rehabilitation sessions or may need additional medical treatment and psychiatric consultation.

### Limitations of the study

- Patients’ compliance to follow up the appointment after feeling better at the last session.
- The issue regards the facility of transportation from rural areas to the tertiary hospital in city offering the CDP as VRT.

## Data Availability

The datasets generated and/or analyzed during the current study are available from the corresponding author on reasonable request.

## Appendix

Diagnostic criteria elaborated by Bárány Society in International Classification of Vestibular Disorders (ICVD) 2017: which defined by criteria A–E below. All five criteria must be fulfilled to make the diagnosis:

A. One or more symptoms of dizziness, unsteadiness, or non-spinning vertigo are present on most days for 3 months or more.
  1. Symptoms last for prolonged (hours long) periods of time, but may wax and wane in severity.
  2. Symptoms need not be present continuously throughout the entire day.
B. Persistent symptoms occur without specific provocation, but are exacerbated by three factors:
  1. Upright posture.
  2. Active or passive motion without regard to direction or position.
  3. Exposure to moving visual stimuli or complex visual patterns.
C. The disorder is precipitated by conditions that cause vertigo, unsteadiness, dizziness, or problems with balance including acute, episodic, or chronic vestibular syndromes, other neurologic or medical illnesses, or psychological distress.
  1. When the precipitant is an acute or episodic condition, symptoms settle into the pattern of criterion A as the precipitant resolves, but they may occur intermittently at first, and then consolidate into a persistent course.
  2. When the precipitant is a chronic syndrome, symptoms may develop slowly at first and worsen gradually.
D. Symptoms cause significant distress or functional impairment.
E. Symptoms are not better accounted for by another disease or disorder.
